# Slow waves on long helices

**DOI:** 10.1038/s41598-022-05345-1

**Published:** 2022-02-03

**Authors:** Lauren E. Barr, Gareth P. Ward, Alastair P. Hibbins, Euan Hendry, J. Roy Sambles

**Affiliations:** Department of Physics and Astronomy, University of Exeter, Exeter, EX4 4QL UK

**Keywords:** Techniques and instrumentation, Slow light, Microwave photonics, Metamaterials

## Abstract

Slowing light in a non-dispersive and controllable fashion opens the door to many new phenomena in photonics. As such, many schemes have been put forward to decrease the velocity of light, most of which are limited in bandwidth or incur high losses. In this paper we show that a long metallic helix supports a low-loss, broadband slow wave with a mode index that can be controlled via geometrical design. For one particular geometry, we characterise the dispersion of the mode, finding a relatively constant mode index of $\sim$ 45 between 10 and 30 GHz. We compare our experimental results to both a geometrical model and full numerical simulation to quantify and understand the limitations in bandwidth. We find that the bandwidth of the region of linear dispersion is associated with the degree of hybridisation between the fields of a helical mode that travels around the helical wire and an axial mode that disperses along the light line. Finally, we discuss approaches to broaden the frequency range of near-constant mode index: we find that placing a straight wire along the axis of the helix suppresses the interaction between the axial and high index modes supported by the helix, leading to both an increase in bandwidth and a more linear dispersion.

## Introduction

‘Slow waves’, electromagnetic waves with a velocity less than the speed of light in the surrounding material^1^, have many applications, including the enhancement of non-linear interactions^2^, manipulation of small particles^3^, increased efficiency of solar cells^4^, sensitive Raman spectroscopy^5^, compact data storage^6^, and in the study of fundamental light-matter interactions^7^. The most extreme cases of slow light have been predicted and measured in a Bose-Einstein condensate with electromagnetically induced transparency^1^, where group velocities of a few metres per second were shown to be attainable. However, the ultra-low temperatures required by these cryogenic systems make them unsuitable for most real-world applications. Solutions based on solid state systems are more practical, such as waveguides encased in negative-index metamaterials^8,9^, or high quality-factor resonances in photonic crystals^10^. Such structured media can be used to control the direction and speed of propagation of electromagnetic waves, and there are many examples of photonic crystal waveguides^11–13^, metamaterials^14^, and metasurfaces^15,16^ that slow the speed of a propagating wave. These systems rely on resonances to achieve slow-light, and hence have narrow operational bandwidths. However, many applications involve the slowing of pulses containing many frequencies, e.g. where slow waves can ease congestion in communication networks by introducing time-delays between coincident signals^17,18^. Hence, increasing the operational bandwidth of slow wave waveguides is of great practical importance^19^, even if at the cost of extreme reductions in speed.

For this purpose, and to offer increased bandwidths, higher-order symmetries in periodic waveguides and surfaces are of great interest, primarily glide (or glide-plane) and screw (or screw-axis) symmetries. In the 1960s and 1970s it was found that waves supported on structures with glide- and screw-symmetry demonstrated a dispersion that crossed at some Brillouin zone boundaries in reciprocal space without forming a band-gap, and hence dispersed almost linearly over a wide frequency range^20^. This analysis, based only on symmetry arguments, was supported by experimental results in the microwave range of the electromagnetic (EM) spectrum^21^ and more complete theoretical studies^22–24^. This catalogue of early work has inspired many more recent investigations into the implications of higher-order symmetries on the dispersion of waves. For example, it has been shown that periodic structures possessing glide-symmetry can be used to create largely dispersionless metasurfaces^25,26^ and wave-guides^27,28^; particularly useful for constructing leaky wave antennas^29,30^, non-dispersive broadband lenses^31,32^, and shields for integrated wave-guides^33^.

Perhaps the simplest structure containing glide symmetry is a helical-wire waveguide^22,23^. By definition, a helix possesses screw symmetry of order $m=\infty$, i.e. it is symmetric under any rotation and corresponding translation, regardless of how small. It has been known for some time that slow waves are supported on helical waveguides^34^ and antennas^35^, and they have become an important component of travelling-wave-tube amplifiers, where the slow speed meant higher amplification of EM waves due to the extended time the wave spent within the amplifying electron beam^36,37^. Recent fabrication improvements have also meant helical structures operating at higher frequencies have become possible, e.g. at terahertz frequencies^38^. However, while it has been shown that the frequency bandwidth of slow waves in helical waveguides and antennas is limited^20,34,35^, there has been no systematic study attempting to characterise, understand and improve upon bandwidth limitations.

In this work we measure the low-loss, broadband slow wave on a metallic helix, with a mode index that can be controlled via geometrical design. For one particular geometry, we characterise the dispersion of the mode, finding a relatively constant mode index of $\sim$ 45 between 10 and 30 GHz. The features in experimentally measured dispersion plots are characterised and explained using both numerical and analytical models. We find that the bandwidth of the near-constant mode index behaviour is constrained by the hybridisation of a high-index helical mode and an axial mode that disperses along the light line. Finally, we find that placing a straight wire at the centre of the helix suppresses the interaction between the axial and high index modes supported by the helix, leading to both an increase in bandwidth and reduced frequency dispersion.

## Experiment

**Figure 1:**
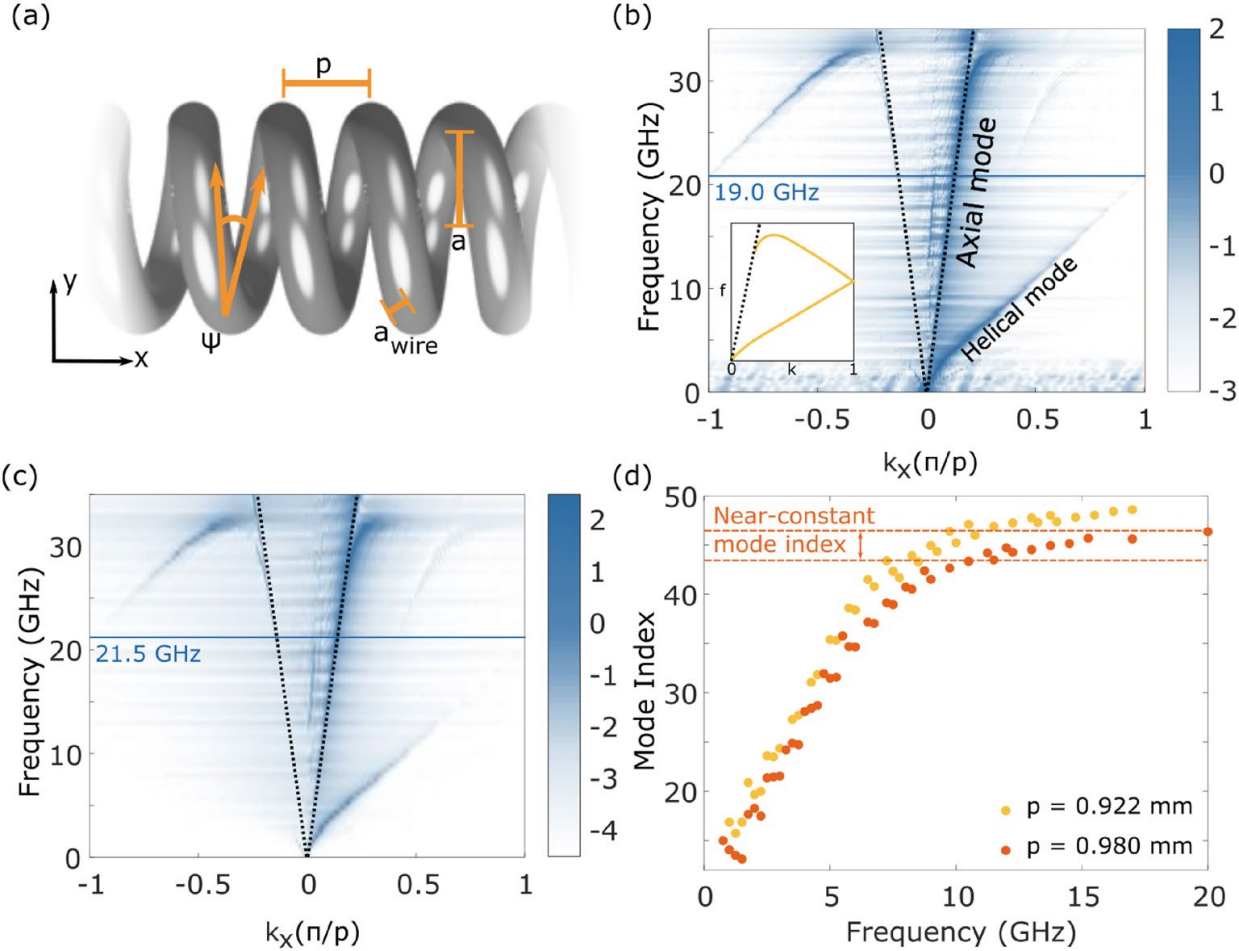
(**a**) Side view of the long helix with the dimensions labelled. (**b**) Experimentally measured dispersion of the wave on a long helix with dimension $\psi = 6.94^{\circ }$, $p = 0.922$ mm and $a = 1.206$ mm. The black dotted line indicates the light line, the axial and helical modes are labelled, and the inset shows a schematic dispersion using the reduced zone scheme. (**c**) Dispersion of the waves on the same helix after it has been stretched, changing the dimensions to $\psi = 7.35^{\circ }$, $p = 0.980$ mm and $a = 1.205$ mm. In both cases the wire diameter is the same, $a_\text {wire} = 0.351$ mm. Blue horizontal lines show the difference in frequencies at which the bands reach the Brillouin zone edge. (**d**) Plot of the mode indices of the waves supported on the two helices, extracted from experimental dispersion of the forward propagating wave. Orange dashed lines highlight the region of near-constant mode index for the longer pitch helix. *Figure made in Inkscape v1.1.1, inkscape.org.*

We experimentally measure the dispersion of the EM wave supported by a long metallic helix by placing a subwavelength near-field antenna close to one end of the helix, and measuring the instantaneous electric field amplitude and phase as a function of frequency and position along the helix using a similar probe antenna. This data is Fourier transformed into reciprocal space, and the dispersion (frequency as a function of propagation constant along the long axis of the helix, $k_\text {x}$) is plotted. Results are shown in Fig. 1. The helix is assumed to be infinitely long, as the ratio of diameter to length is around $2 \times 10^{-4}$, making the end-effects negligible, and the self-resonance frequency associated with the total length of wire in the helix^39^ very low ($\sim 0.01$ Hz). The intensity of the signal measured depends on the coupling between the eigenmode of the helix and the source, and similarly between the supported mode and the probe. Due to the measurement configuration, the data in Fig. 1b,c show that only waves with positive group velocity are detected. Waves with negative group velocity, as defined with respect to the helix axis, can be detected in a separate measurement, by moving the source to the other end of the waveguide and scanning the detector in the opposite direction. For clarity, we provide a schematic of the dispersion of the helical mode using the reduced zone scheme in the inset of Fig. 1b.

The dispersion relations of waves supported by two different helices are presented in Fig. 1, where the helix in (b) has a shorter pitch than that in (c). In fact, the latter is simply a stretched version of the former, where the dimensions for both are given in the figure caption. In each case two modes are detected—one that disperses along the light line with an index determined by the surrounding medium (air in this case): this is referred to as the ‘axial’ mode. A higher index mode is also seen, which is termed the ‘helical’ mode. The additional faint band seen just beyond the axial mode in both cases is an artefact of the measurement technique, caused by the presence of the source and detector.

For both helices the helical mode wave disperses linearly over a wide frequency range, and no band-gap forms at the edge of the first Brillouin zone (BZ): these are characteristics of systems with screw symmetry^22,23^. The lack of band-gap is due to the fact that we define the BZ according to the unit cell defined by the translation symmetry of the helix. This unit cell is the smallest section from which the rest of the structure can be built by translating it along an axis, as described by the translation operator, **T**,

1 $$\begin{aligned} \mathbf{T} = {\left\{ \begin{array}{ll} \begin{matrix} x &{} \rightarrow &{} x+a \\ y &{} \rightarrow &{} y .\\ \end{matrix} \end{array}\right. } \end{aligned}$$

In this case *a* is the length of one turn of the helix. However, this unit cell can be reduced further by using the *m*-order screw symmetry operator, $\mathbf{S} _\text {m}$,

2 $$\begin{aligned} \mathbf{S} _\text {m} = {\left\{ \begin{array}{ll} \begin{matrix} x &{} \rightarrow &{} x + \frac{a}{m} \\ \theta &{} \rightarrow &{} \theta + \frac{2\pi }{m} .\\ \end{matrix} \end{array}\right. } \end{aligned}$$

The screw symmetry unit cell of a *m*-order screw symmetric system is *m* times smaller than the translation symmetry unit cell of the same system. In reciprocal space the BZ will be *m* times larger. In the case of the helix, $m=\infty$, and it is not practical to plot an infinitely large BZ. Instead we represent the band structure of a metasurface using the reduced-zone scheme, where all bands lying outside the first (translation symmetry) BZ are folded back into the first.

The wave of the helical mode of the short pitch helix, Fig. 1b, crosses the BZ boundary at 21.5 GHz, whereas the wave on the long pitch helix (c) crosses at 19.0 GHz. We can better consider this difference by studying the frequency dependence of the mode index *n*, (Fig. 1d) which is extracted from the Fourier amplitude plots, (b) and (c). The mode index we discuss throughout the paper describes the phase velocity of a wave along the helix, analogous to the refractive index: $n = c/v_{\text {p}}$ and $v_{\text {p}} = \omega /k_\text {x}$. The most compelling feature of (d) is the region of slowly-varying mode index above 8 GHz. The mode indices achieved here are extremely high at around 45, far exceeding those of naturally occurring materials and rivalling all but the most extreme indices found in artificial media^40^. As the wave disperses linearly at high frequencies the group index is equal to the mode index, since $\text {d}n/\text {d}k_\text {x} = 0$. This means the group velocity of the wave is also slowed by a factor of 45 by the longer pitch helix, compared to a wave in free space. While this is by no means the slowest wave reported^1^, the range of frequencies over which the mode index is high is large—we describe the mode index as near constant when the variation is less than $\pm 3\%$ of the central mode index, as marked by the dashed lines in Fig. 1d. Helices also provide an advantage over traditional slow wave waveguides in their tunability through geometry. Comparing the mode indices of the two helices at 17 GHz, there is a difference in index of around 3. This was achieved by a stretch of only 6% in the helix. With sufficiently careful measurements, flexible helices can also offer a route to experimentally explore broadband non-linear effects in microwave metamaterials^41,42^.

## Theoretical descriptions

We now use three approaches to theoretically describe the wave supported by the long helix: a numerical simulation, a geometrical approximation, and an analytical description. These models provide the insight needed to understand, and subsequently extend, the bandwidth of the high index mode.

### Numerical model

**Figure 2:**
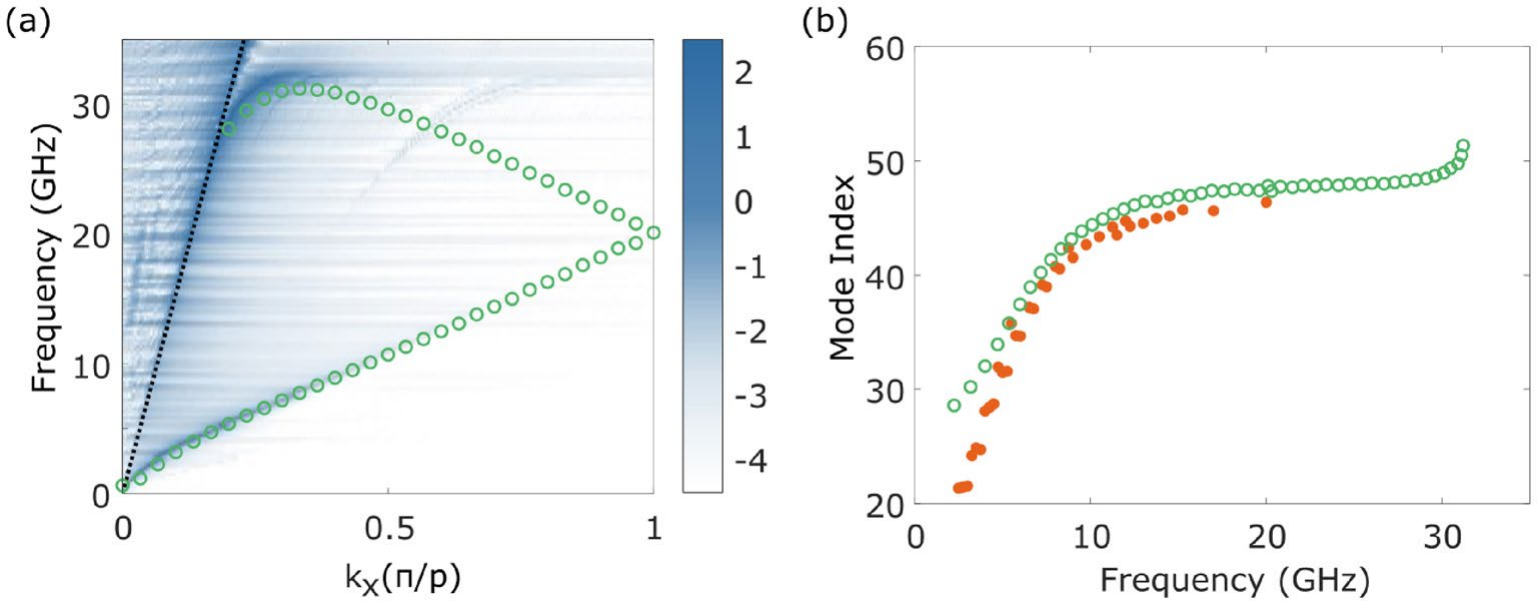
(**a**) Experimentally measured dispersion (blue scale) and the dispersion calculated from a finite element method (FEM) model (green empty circles). The black dotted line represents the light line. (**b**) Mode index extracted from the experiment (orange filled circles) and from the FEM model (green empty circles). In both cases the dimensions of the helix are those described for the helix in Fig. 1c.

A finite element method (FEM) model, created using COMSOL Multiphysics software, offers further insight into the experimental phenomena observed. By studying plots of the electric field and current, we can arrive at an intuitive interpretation of the linear dispersion. The model uses Floquet periodic boundary conditions that assume an infinitely repeated unit cell of one turn of the helix, and hence the eigenmode dispersion is plotted using the reduced zone representation. Furthermore, in the model the complexity introduced by coupling between probe and bound wave in the experiment is removed. The agreement between the modelled and experimental dispersion is good, as shown in Fig. 2a. Small inconsistencies arise from the uncertainty in the measurement of the helix radius, and the slightly inhomogeneous stretching of the helix. The mode index extracted from the FEM model also agrees well with the experimental results in Fig. 2b. However at very low frequencies the FEM model becomes unreliable due to the wavelength-pitch ratio, and modes that are within the light-line are artificially perturbed.

Plots of the simulated surface current density on the helix at various points along the dispersion can be found in the supplementary material. These show that the current distribution is periodic along the helix axis. As the wavevector increases, more wavelengths are supported within each turn of the helix. Since the wire of the helix is continuous, there is no condition that restricts the wavelengths to be any function of the dimension. Therefore we can expect the slow-wave behaviour to be supported over a very broad frequency range. There remains a limit to the frequency range over which the mode index is almost constant, and the factors that cause the dispersion to stray from the ideal linear case will be discussed in the final section of the paper.

### Geometrical approximation

**Figure 3:**
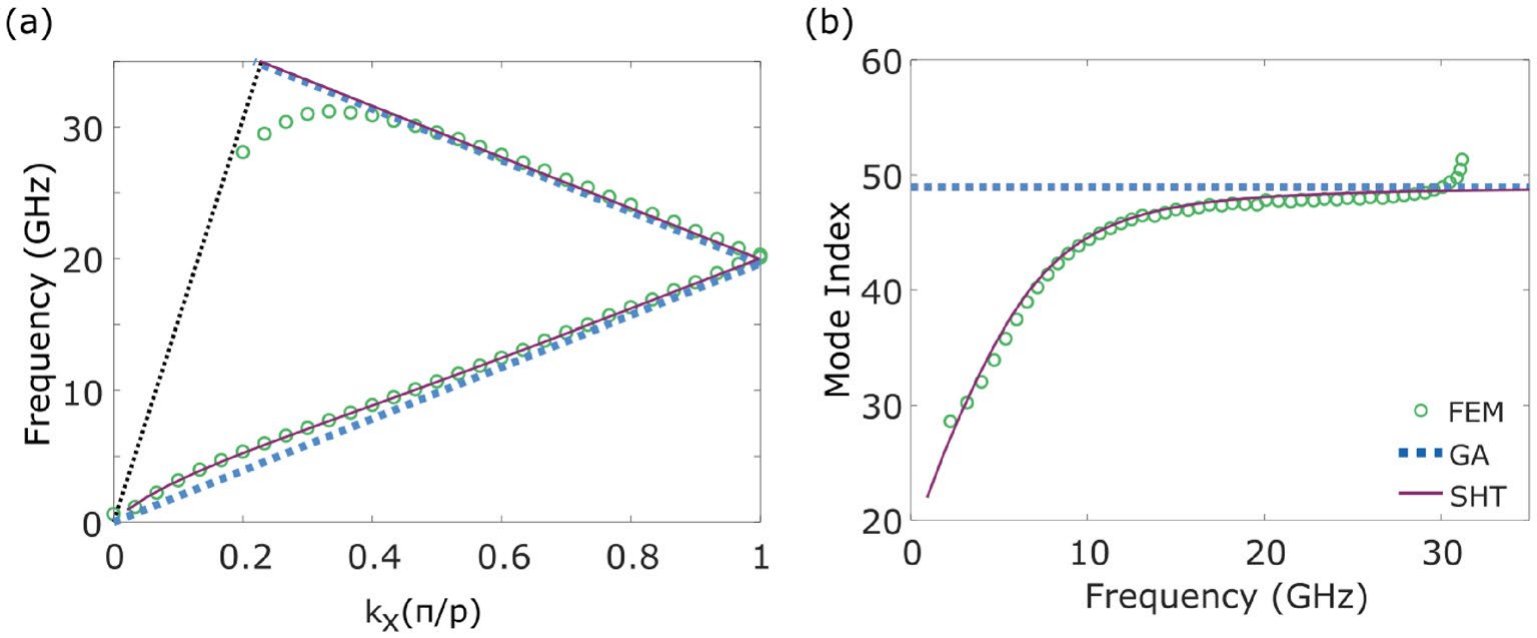
(**a**) Dispersion of the wave on a long helix with dimensions of that in Fig. 1c, found from a finite element method (FEM) model (green empty circles), a geometrical approximation (blue dotted line) and the sheath helix theory (pink solid line). (**b**) Mode index extracted from the dispersion from the FEM model (green empty circles), geometrical approximation (blue dotted line) and sheath helix theory (pink solid line).

The plots of surface current given in the supplementary material also show that the current along the helical wire is strongest at the points where the wires in adjacent turns are closest. This implies that the currents, and therefore the wave, follow a path along the length of the coiled up wire in the helix. This assumption can be used to derive a geometrical approximation (GA) for the dispersion of the wave. For a perfectly electrically conducting straight wire the wave would travel at the speed of light in the surrounding medium (air in this case)^43^, and this is a valid approximation at GHz frequencies. Bending the wire into a helical shape increases the length of the path the wave must travel. The dispersion of the wave, and subsequently the mode index, can be naively calculated by finding the ratio between the distances covered when travelling around the wire or straight along the axis of the helix, via the equation:

3 $$\begin{aligned} k_\text {x}^\text {geom} = k_0 \frac{\root \of {p^2 + 4 \pi ^2 a^2}}{p} \end{aligned}$$

where *a* and *p* are as defined in Fig. 1a. A comparison between the results of the FEM model (circles) and the geometrical approximation (dotted lines) is shown in Fig. 3. Both the dispersion in (a) and the mode index in (b) are predicted well by the geometrical approximation for moderate wave-vectors. The agreement between modelling and geometrical approximation at these wave-vectors increases as the thickness of the wire decreases and the wave is more confined to travel along only this path, rather than propagate along different parts of a thicker wire surface. This is a useful approximation when designing high index media, and can be used to select the geometry required for a given index. However, as we will show in the next section, it is only valid in the frequency range where the dispersion is linear and the mode index is near-constant, even for infinitely thin wires. The mode index found using this method is referred to as the asymptotic mode index.

### Analytical calculation

A more accurate method for finding the dispersion of the wave on a helix is through an analytical model, such as the sheath helix theory (SHT)^34^, which takes into account the decay of electric fields towards the centre of the helix and directly out from the helix. It is assumed that the helix can be approximated by an infinitely thin conducting cylinder with an anisotropic impedance. These assumptions reduce the accuracy of the theory slightly, and more recent works have built upon these to include the width of the wire, and the coupling of adjacent turns of helices^39^. However this model is sufficient to describe the low frequency behaviour of the wave bound to a long helix, as illustrated by comparing the results (solid lines) to those of an FEM model (empty circles) in Fig. 3a,b.

The waves inside the helix are described by modified Bessel functions of the first kind ($I_\text {q}$) of order q and argument $(\gamma a)$, while the fields outside the helix are described by modified Bessel functions of the second kind ($K_\text {q}$). This is to ensure that the fields outside the helix decay with increasing distance from the helix, and that inside they grow as they approach the helix. $\gamma$ is a relative wave-vector, and is defined as

4 $$\begin{aligned} \gamma ^2 = k_\text {x}^2 - k_0^2. \end{aligned}$$

A full derivation of the dispersion relation is provided by Sensiper et al.^34^, where the determinantal equation according to the sheath helix theory, is

5 $$\begin{aligned} \frac{I'_\text {q} (\gamma a) K'_\text {q} (\gamma a)}{I_\text {q} (\gamma a) K_\text {q} (\gamma a)} = \frac{\left( \gamma ^2 \text {a}^2 + k_\text {x} q a \text {cot} \psi \right) ^2}{k_0^2 a^2 \gamma ^2 a^2 \text {cot}^2 \psi }, \end{aligned}$$

where the prime signifies differentiation with respect to the argument, and *a*, *p* and $\psi$ are as defined in Fig. 1a. For the $q=0$ mode this simplifies to

6 $$\begin{aligned} \frac{I_1 (\gamma a) K_1 (\gamma a)}{I_0 (\gamma a) K_0 (\gamma a)} = \frac{\gamma ^2}{k_0^2 \text {cot}^2 \psi }. \end{aligned}$$

Taking the low frequency limit of this equation confirms that at zero frequency the wave will travel along the helix at the speed of light in the surrounding material. Similarly, the high frequency limit of this dispersion relation agrees with the geometrical approximation where the mode simply follows the path of the helix (Eq. 3). Starting in the low-frequency limit, as $\gamma a$ increases, the dispersion of the wave will turn towards the linear region with a curvature dependent on the argument of the Bessel functions. Since these describe the fields inside and outside the helix, we surmise that the curvature of the dispersion in this region is determined by the decay of the fields in relation to the radius of the helix. In fact this will be shown later in the paper.

## Limits to the bandwidth

We use the understanding of the behaviour of the helical mode gained in the previous section to study the factors that limit the bandwidth of the high index mode. We then use this insight to propose a route to increasing the bandwidth: placing a conducting wire at the centre of the helix to manipulate the electric fields.

### Low frequency limit

**Figure 4:**
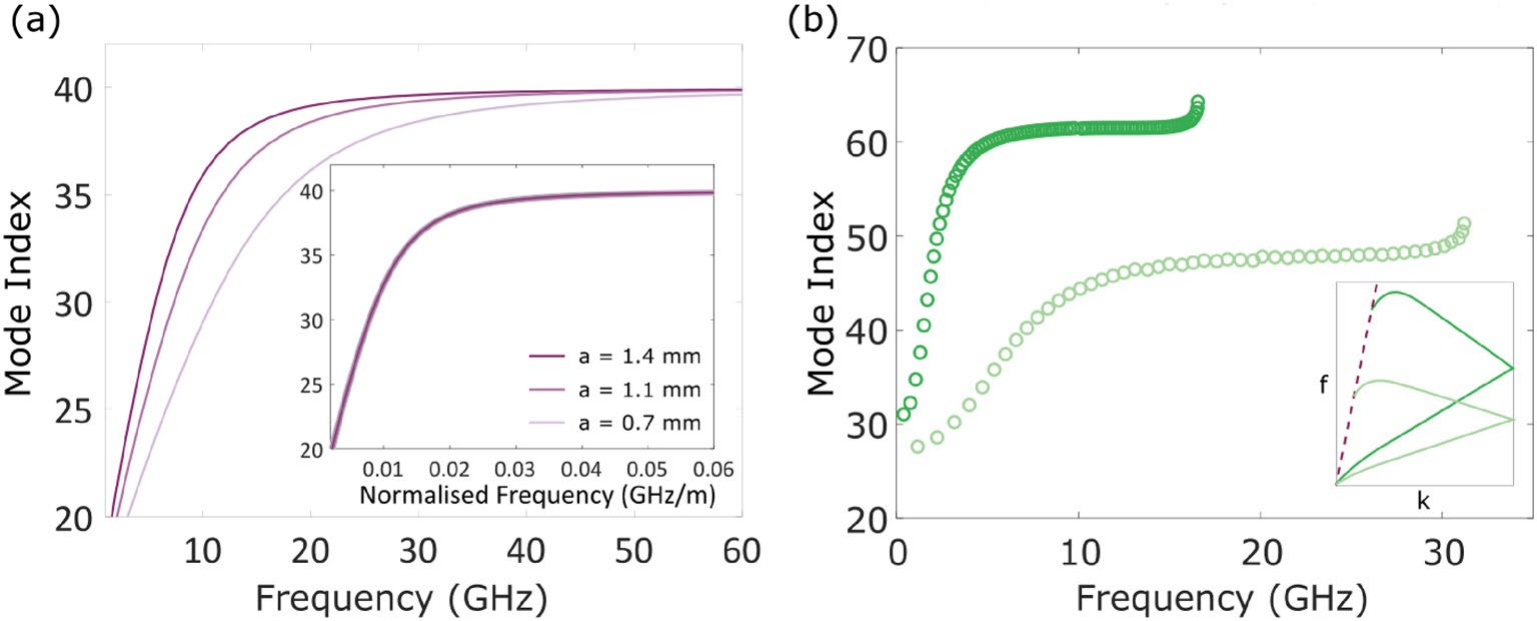
(**a**) Analytical mode index for three different dimensions of helices, chosen to support a wave with the same asymptotic mode index, i.e. $\psi = 9.04^\circ$ and $a = p$ for all helices. The inset in (**a**) shows the same mode indices as a function of normalised frequency, $f_\text {norm} = f\times a$, where the lines perfectly overlap. (**b**) Mode index of the wave in helix (**c**) of Fig. 1 in light green, and a helix with dimensions $\psi = 5.83^{\circ }$, $p = 1.54$ mm, $a = 2.4$ mm and $a_\text {wire} = 0.7$ mm in dark green. The larger index means a lower high-frequency limit to the near-constant index, as illustrated by the inset schematic dispersions.

Good agreement is seen in Fig. 3 between dispersion relations found from the sheath helix theory (solid lines) and the FEM model (empty circles) at low frequencies. This theory can be used to probe the low frequency behaviour of the bound wave for various dimensions of helices. Figure 4a illustrates how the mode index depends on the helix geometry at low frequencies. Each line is the mode index of the wave supported on a helix chosen to have the same asymptotic mode index. The darkest pink line corresponds to the helix with the largest pitch and helix radius, and the lightest pink line, the helix with the smallest pitch and helix radius. The inset in Fig. 4a shows the mode indices of the same helices, where the frequency scale has been normalised to the helix radius, $f_\text {norm} = f\times a$. The frequency at which the bound wave on the helix approaches the asymptotic limit is proportional to 1/*a*, so on this normalised frequency scale the mode indices perfectly overlap. This supports the argument put forward based on the sheath helix theory—the curvature of the dispersion at low wave-vectors is determined by the decay length of the fields around the helices compared to the helix radius.

It also highlights the origins of the low frequency limit to the bandwidth of a near-constant mode index. This limit can be somewhat ameliorated by making the helix as large as possible, so that even at low frequencies the decay of the fields is over a small length scale compared to the helix dimensions. However, practical implementations may result in limitation in size that cannot be avoided. For such circumstances, we discuss an alternative route to overcoming this problem in the final section of this paper.

### High frequency limit

There is also a high frequency limit to the region of near-constant mode index. This is not taken into account in either the geometrical approximation or the theoretical calculations, but is clearly seen in the experimental (Fig. 1) and FEM dispersion relations (Fig. 2). The limit is a consequence of interaction of the helical mode with the axial mode, which disperses close to the light line. The axial mode is essentially a wave that travels along the major axis of the helix without perturbation from the structure of the helix.

Both axial and helical modes exist along the helix within the first BZ, with different dispersion relations. The frequency and wave-vector of both modes match at a point where the two waves are travelling in opposite directions along the helix. The waves will hybridise to produce a standing wave with zero group velocity. The mode index also increases accordingly at this point.

This limit is not so straightforward to address, as tuning the overall size of the helix will not help. In Fig. 4b the mode indices of two different helices are shown as a function of frequency. It is clear that the frequency at which the region of linear index ends is inversely proportional to the index. This is because the axial wave will disperse in the same manner on all helices (in air), but a helical wave with a higher index will have a dispersion with a larger gradient, and so will meet the axial wave at a lower frequency on the dispersion diagram. This is illustrated in the schematic inset in Fig. 3b.

## Increasing the bandwidth

The previous sections have shown that the bandwidth of the near-constant index is dependent on different factors at the high and low frequency limit. The high frequency limit is determined by hybridisation of the axial and helical mode. At low frequencies the decay of fields around the helix must be over a short distance compared to the radius of the helix to achieve a constant index. Equipped with this knowledge, we suggest a modification to the geometry that increases the bandwidth through manipulation of the fields—inserting a straight wire into the centre of the helix. A cylindrical wire in the centre does not alter the symmetry of the system. This modification will also leave the helix open so that the wave can be used to probe non-linear and enhanced interactions, and allow the measurement technique used here. However, the presence of the wire will perturb the evanescent fields within the helix, and lead to an increase in bandwidth as discussed in more detail below.

**Figure 5:**
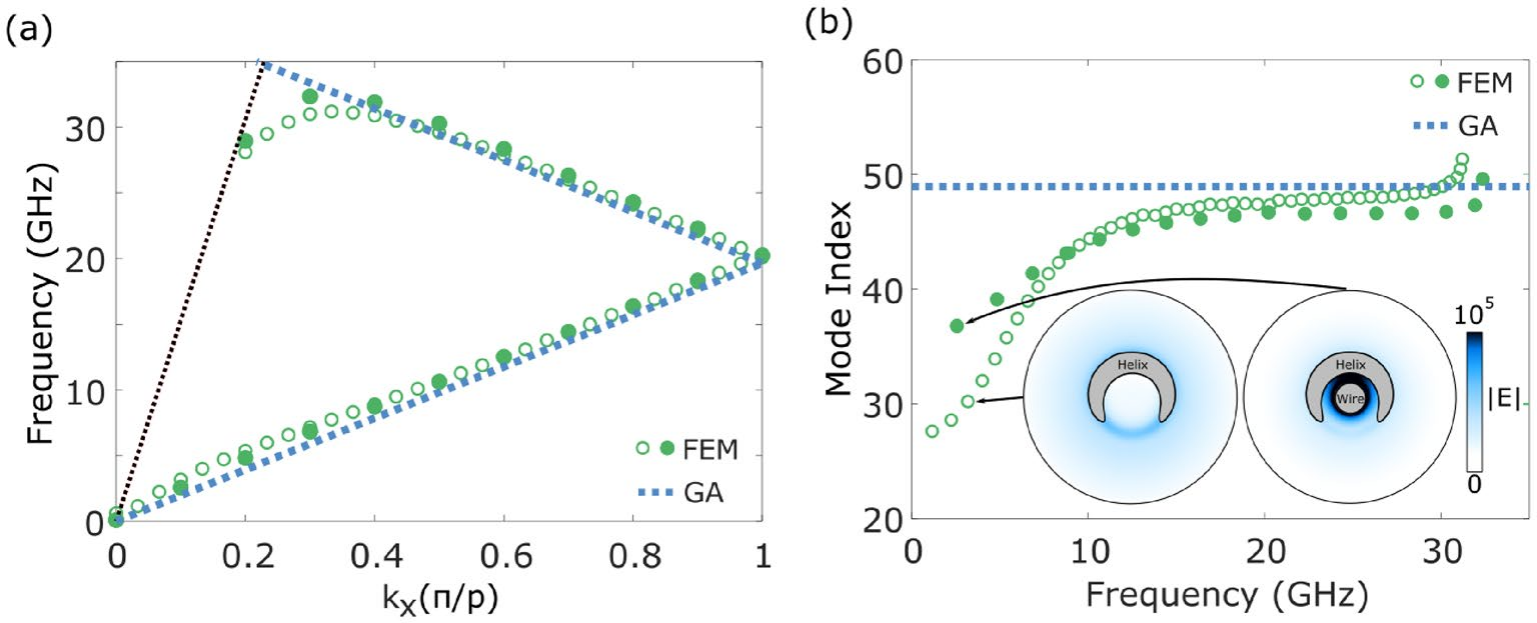
Dispersion (**a**) and mode index (**b**) of a helix with dimensions described in Fig. 1c. The black dotted line in (**a**) represents the light line. Blue dotted lines are results from the geometrical approximation (GA), green empty circles are from a finite element method (FEM) model of an empty helix, and green filled circles are from a FEM model of a helix with a cylindrical wire placed in the centre, where the wire diameter is $1.5 \times$ that of the wire in the helix. Inset in (**b**) are cross-sections of the time-averaged electric field through the empty helix (left) and the helix with the central wire (right) at $k_\text {x} = 0.1$. *Figure made in Inkscape v1.1.1, inkscape.org.*

Inset in Fig. 5b are two plots of the time-averaged electric field intensity at cross-sections, perpendicular to the helix axis, through an empty helix (left) and a helix with a wire in the centre (right) at the same low wave-vector. When the centre of the helix is empty the electric field is strongest between adjacent turns of the helix, but decays over quite a large distance from the helical wire. When a wire is present in the centre of the helix the electric field is more strongly confined to the helix, particularly to the centre. The wire imposes new boundary conditions on the fields at the centre, so that the tangential electric field and the normal magnetic field must both be zero. This forces the fields to decay over a shorter length scale. According to the conclusions drawn so far, this will lead to a more linear dispersion at low frequencies, as seen in Fig. 5a. The dispersion of the wave on the helix with the central wire (filled green circles) tends more towards the geometrical approximation (dotted blue line) than the empty helix (empty green circles) at both low and high wave-vectors. For wave-vectors between around $0.4\pi /p$ and $1.4\pi /p$ the dispersion does not change significantly with the addition of the central wire, as most of the electric field is concentrated around the wire of the helix. The mode index for a helix with a central wire, shown in Fig. 5b, does not reach such low values at low frequencies, as expected from the distribution of the fields.

The presence of the central wire also increases the upper frequency limit. As shown in Fig. 5b, the region of near-constant index extends to frequencies around 2 GHz higher than that of the empty helix. The axial mode is still present, so the axial and helical modes still hybridise, but the extent in wave-vector over which the interaction occurs is smaller. This can be explained by considering that the confinement of the fields is greater when the central wire is present. This causes the mode overlap between axial and helical modes at wave-vectors slightly away from the point of matched frequency to decrease, and the interaction of the modes becomes weaker. The interaction of the modes decreases further as the radius of the central wire increases, as presented in the supplementary material, until the point where the central wire makes contact with the helix.

The addition of a central wire provides a new degree of freedom that can be tuned to give a greater range of properties. It does not introduce band-gaps, but if it is moved closer to one side, the order of screw symmetry will be reduced to $m=1$, and a band-gap will open at the edge of the BZ. One can now imagine replacing the straight central wire with a periodically varying one, for example a sinusoid, saw-tooth or square wave. If this periodicity is on a length scale much larger than the pitch of the helix and chosen carefully, it is possible to create a wave-guide with a varying mode index based on broken symmetries. There are, however, certain applications where the addition of this central wire is not useful, for example in travelling wave-tube amplifiers. In such cases, the geometric considerations discussed in the previous section will be most important for achieving large bandwidths.

## Conclusions

In this paper, the electromagnetic waves supported on an infinitely long helix have been investigated. Two modes were measured in the experiment—an axial mode that dispersed along the light line, and a helical mode with a high mode index.

Due to the screw symmetry of the helix, no band-gap was seen in the helical mode at the edge of the Brillouin zone, and the dispersion showed a large region of near-constant mode index. This asymptotic mode index is predicted by a geometrical model based on the length of the wire in one turn of the helix. A more tightly wound helix, with a larger radius or shorter pitch, produced a higher index, and this can be tuned by stretching or compressing the helix.

The origins of the upper and lower frequency limits to this region of near-constant index were then investigated. From the results of a sheath helix analytical theory, it was found that the lower frequency limit was inversely proportional to the helix dimension, and a larger overall helix will decrease the frequency at which the dispersion becomes linear. The upper frequency limit was set by the hybridisation of the axial and helical modes on the helix, and was found to be inversely proportional to the mode index.

Finally, the addition of a straight wire in the centre of the helix was studied. It was found that this addition improved the linearity of the dispersion by forcing a faster decay in the electric fields inside the helix, and causing the dispersion to tend towards the asymptotic limit at lower frequencies. The tighter confinement of the fields to the helix also weakened the interaction between the axial and helical modes, increasing the upper limiting frequency at the same time.

## Methods

### FEM modelling

The finite element method modelling presented here was performed with Comsol Multiphysics. All metal objects were modelled as perfectly electrically conducting boundaries, a good approximation at microwave frequencies. Floquet periodic conditions were set on the faces intersecting the helix to create an infinitely long helix. A perfectly matched layer was placed around the outside to absorb scattered fields. The eigenvalue solver was used to find the eigenfrequencies of the system as a function of wave vector along the major helix axis.

### Experimental techniques

The helix measured was re-purposed from a traditional net-curtain rail and dimensions carefully measured. During the measurement it was suspended at either end by wooden frame and held by lead weights to ensure it was straight and the pitch was consistent. A vector network analyser was used to send a microwave signal to a near-field source (short, straight length of stripped coaxial cable) that was placed at one end of the helix. Another similar antenna was used as a probe, and scanned along the length of the helix. A linear array of measurements in space of the complex electric field measured by the probe was Fourier transformed from real-space to reciprocal-space to find the dispersion.

## Supplementary Information

Supplementary Information.
