# Supplementary material for: Slow waves on long helices
Source: Sci Rep. 2022 Feb 3;12:1902. doi: 10.1038/s41598-022-05345-1 (PMC8814024; doi:10.1038/s41598-022-05345-1)
Supplement: Supplementary file 1 — Supplementary Information. [file 41598_2022_5345_MOESM1_ESM.pdf]

Slow waves on long helices - Supplementary information

Lauren E. Barr, Gareth P. Ward,
Alastair P. Hibbins, Euan Hendry & J. Roy Sambles

December 23, 2021

1 Introduction

This document contains supplementary information for the paper *Slowing Waves on Long Helices*. The first section contains plots of the surface current around the wires of the helix taken from the finite element method model. The second section contains plots of the dispersion relation and the mode index for helices with different central wires, also found using finite element method modelling.

2 Surface current density

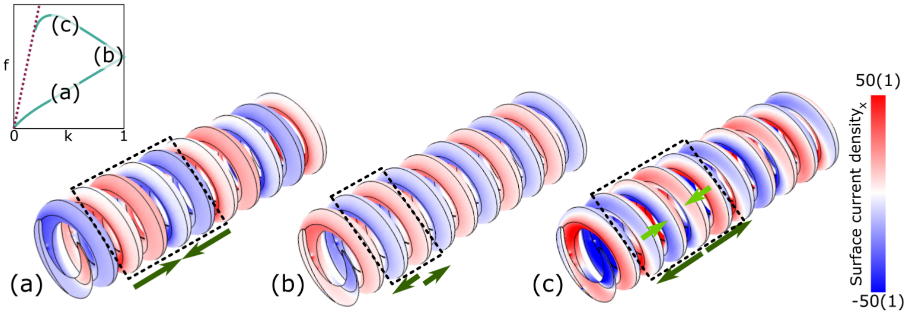

Figure S 1. Plots of surface current density directed along the helix axis for helices of dimensions: pitch, $\psi = 7.35°$; period, $p = 0.980$ mm; helix radius, $a = 1.205$ mm; and wire radius, $a_{\mathrm{wire}} = 0.351$ mm. The inset shows the positions on the dispersion diagram where (a), (b) and (c) were calculated. Black dashed boxes highlight one repeating unit in the surface current, and green arrows indicate the direction along the helix axis of the surface current within the repeating unit. *Figure made in Inkscape v1.1.1, inkscape.org.*

Figure S 1 (a), (b) and (c) show the components of the surface current density along the helix axis at different wave-vectors. In Figure S 1 (a), $k_x = 0.5$ and

the frequency is that of the mode in the lowest band. The surface current density is periodic along the helix axis, with the dashed black box surrounding one repeating unit of four turns. The direction of the surface current density is indicated by the dark green arrows. The largest surface current density is found at the points on the wire where they are closest to the wire of the adjacent turn. For each turn there are two regions where the currents are strongest. This differs from the case of a straight wire where the surface current density would be constant around the circumference of the wire.

Figure S 1 (b) shows the surface current density at the Brillouin Zone (BZ) edge, $k_{\mathrm{x}} = 1$. The frequency at this point meets the condition for a half-wavelength to fit inside one turn of the wire. In fact, the repeating unit in the current spans across only two turns of the wire, shown by the black dashed box.

In Figure S 1 (c), we consider the $k_{\mathrm{x}} = 0.5$ solution in the upper band of the dispersion plot. The repeating unit contains four turns of the helix, as in (a), although now the current distribution is more complicated as the wavelength is smaller than the length of wire in one turn of the helix, as shown by the green arrows that depict the direction of the surface current in the repeating unit.

These plots show that the current distribution is periodic along the helix axis. At higher wavevectors more wavelengths are supported within each turn of the helix, but since the wire of the helix is continuous there is no condition that restricts the wavelengths to be the function of a dimension, as in a resonant system.

3 Varying the radius of the central wire

In the main manuscript we describe the effects of placing a straight wire in the centre of the helix. We explain how this central wire forces the electric field within the helix to decay over a shorter distance, thus decreasing the interaction between the axial and helical modes, and increasing the bandwidth. Here we present the results of finite element method simulations where the radius of the central wire varies, but remains smaller than the inner radius of the helix.

Figure S 3 shows the results of finite element method modelling where the radius of the central wire increases from $0.5 \times a_{\mathrm{wire}}$ to $2.0 \times a_{\mathrm{wire}}$. Minimal changes are observed in the dispersion diagrams, (a), (b) and (c). However when the mode indices are calculated for each helix-wire system, shown in Figure S 3 (d), we see that the region over which the mode index is linear with frequency increases as the radius of the central wire increases. This further supports our hypothesis that the manipulation of the fields within the helix leads to a decreased interaction between modes and an increased bandwidth.

It can also be seen from Figure S 3 (d) that increasing the central wire radius decreases the mode index overall. This is not yet well understood, but it is clear that there is another compromise being made when extending the bandwidth of slow waves on long helices, and further investigations are needed to understand how this may be overcome.

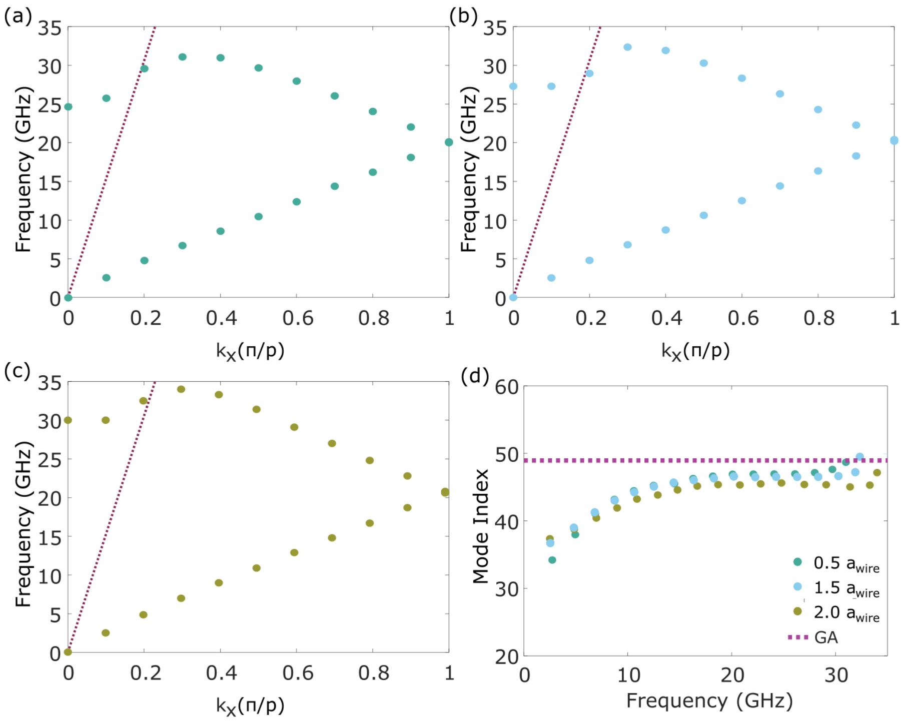

Figure S 2. (a), (b) and (c) show the dispersion diagrams of modes bound to helices with dimensions: pitch, $\psi = 7.35°$; period, $p = 0.980$ mm; helix radius, $a = 1.205$ mm; and wire radius, $a_{\text{wire}} = 0.351$ mm, with a central cylindrical wire in each with radii of 0.5, 1.5 and 2.0 $\times a_{\text{wire}}$, respectively. The red dotted line indicates the light line. (d) shows the mode indices for each helix-wire system, with the purple dashed line showing the results of the geometrical approximation.

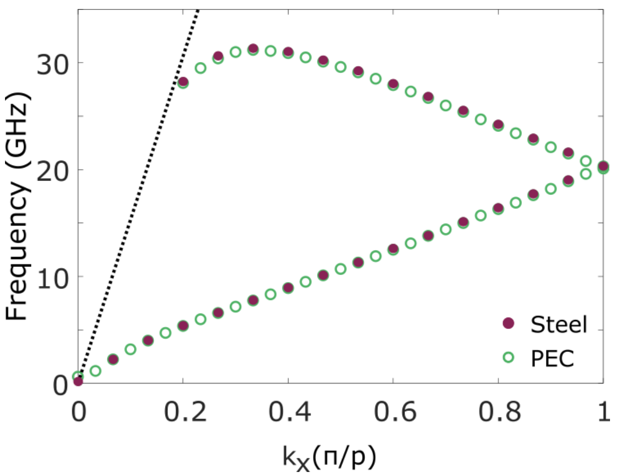

Figure S 3. Simulated dispersion diagram of the modes on a metallic helix with dimensions the same as those used in the main text ($\psi = 7.35°$, $p = 0.980$ mm and $a = 1.205$ mm). The black dotted line represents the light line, green empty circles are the results when the metal is described as a perfect electric conductor (PEC) and red filled circles show the dispersion when the metal has the impedance of steel.

4 Conductivity of real metal

In this work we treat the metal of the helix as a perfect electric conductor (PEC), although it is actually made of steel and has a finite conductivity. At the frequencies we study in this work (around $\times 10^{10}$ Hz), this is a good approximation as the electric field does not decay far into the metal and we are well below the plasma frequency (on the order of $\times 10^{16}$ Hz).

To verify the validity of this approximation, we compare the dispersion relation found from finite element simulations where the metal is represented by a PEC boundary layer and a boundary layer with the impedance of steel. In both cases we assume there is no penetration of the electromagnetic field into the metal in order to reduce the computation time required.

It can be seen that there is very little difference between the dispersion relations found assuming a PEC metal or steel, which shows that our approximation is valid and our model represents the experimental conditions well. The finite conductivity has very slightly increased the steepness of the dispersion of the helical mode, meaning the mode index will be slightly lower. Therefore, in regimes where the PEC approximation is not valid, a large conductivity will be needed to achieve high index modes.
